# Population Dynamics and Diet of the Madamango Sea Catfish *Cathorops spixii* (Agassiz, 1829) (Siluriformes: Ariidae) in a Tropical Bight in Southeastern Brazil

**DOI:** 10.1371/journal.pone.0081257

**Published:** 2013-11-25

**Authors:** Márcia Denadai, Maíra Pombo, Flávia Borges Santos, Eduardo Bessa, Adriana Ferreira, Alexander Turra

**Affiliations:** 1 Centro Universitário Módulo. Caraguatatuba, São Paulo, Brazil; 2 Instituto Oceanográfico da Universidade de São Paulo, Departamento de Oceanografia Biológica, São Paulo, São Paulo, Brazil; 3 Universidade Estadual do Sudoeste da Bahia, Departamento de Ciências Naturais, Vitória da Conquista, Bahia, Brazil; 4 Universidade do Estado de Mato Grosso, Laboratório de Ecologia Comportamental da Reprodução, Tangara da Serra, Mato Grosso, Brazil; 5 Centro Universitário da Fundação de Ensino Octávio Bastos. São João da Boa Vista, São Paulo, Brazil; Universidade Federal do Rio de Janeiro, Brazil

## Abstract

The madamango sea catfish, *Cathorops spixii* (Siluriformes: Ariidae), is often among the most abundant fishes on the South American Atlantic coast. In the present study, conducted in shallow, non-estuarine coastal areas of Caraguatatuba Bight in southeastern Brazil, collections of this species, the most abundant member of the ichthyofauna, included primarily medium-sized individuals, indicating that the area may play a specific role in their development. Although studies of the local ichthyofauna have been much neglected, the area is economically important and its ecological significance is undervalued. This study primarily treats habitat use by *C. spixii*, assessing certain population parameters and the dietary composition. Monthly samples were taken from August 2003 through October 2004, with three trawls in two areas, corresponding to depths of about 1 to 4 m. The catfish showed two main peaks of abundance in the area, in April/May and July 2004. A mode around 9 cm SL persisted through time, and the entrance of younger recruits peaked from January to April. The low estimate for body-growth parameters (K = 0.16) corroborates some K-strategist characteristics of the species. The asymptotic length was 27.3 cm SL and total mortality (Z) was 1.01 yr^−1^. *Cathorops spixii* showed an omnivorous feeding habit, preying mainly upon polychaetes, copepods and bivalves, with considerable seasonality in its diet.

## Introduction

Members of the order Siluriformes, popularly known as catfishes, show a complex geographic distribution throughout the world, and one or more species are often considerably abundant in a community [1]. Among this primarily freshwater group, the family Ariidae is marine, and its members also show several adaptations, which include mouthbreeding [2], [3]. This group is less studied than its freshwater relatives. The ariid *Cathorops spixii* is frequently the most abundant fish in coastal communities of the southeast Atlantic [4]–[7]. Perhaps because of its abundance, recently the species has been widely used in attempts to indicate the concentration of pollutants [8]–[10]. Although many studies have included some features of this species, knowledge about its ecological aspects is mostly included in community studies, and mainly from estuarine regions [7], [11]–[15]. Most of the specifically ecological studies on the species or family deal with reproduction [16]–[20], and those on habitat use were, again, conducted primarily in estuaries [14], [21], [22], as were studies on diet [19], [23], [24].

The madamango sea catfish *Cathorops spixii* inhabits shallow coastal waters, from lagoons and river mouths to hypersaline waters. It is generally accepted that ariids use estuaries as spawning and nursery areas, and outer areas for feeding [25]. They prey mainly upon the zoobenthos, especially crustaceans and polychaetes, and commonly also consume small fish [26], [27]. Corroborating some of their K-strategist characteristics (well-developed parental care, few and large eggs), *C. spixii* shows slow growth, with the largest individuals reaching around 30 cm in total length.

In the present study, *C. spixii* was the most abundant species of 61 caught from August 2003 through October 2004 (data not shown), totaling 6072 individuals, and comprising 27.8% of the total catch in the period. Similarly, *C. spixii* was also the most abundant fish in other coastal studies in the western Atlantic. This species is potentially subject to overexploitation caused by the shrimp fishery, and to the many other human impacts occurring in these areas. Therefore, even though *C. spixii* has a low economic value, population studies are needed to develop knowledge of the species and its habitat areas, and to implement effective management of the habitats.

This study was performed in shallow areas of the tropical Caraguatatuba Bight, southeastern Brazil, which although it is often called a “bay” is actually a relatively exposed bight. These coastal areas are little influenced by continental waters, and therefore are an unusual site for studies on the population biology of this species. The local ichthyofauna has been little studied, even though the area is economically important. The present study assessed environmental factors in the bight in relation to general aspects of the population ecology of *C. spixii*, including its spatio-temporal distribution, abundance and size, body growth parameters, and diet.

## Methods

### Study area

Caraguatatuba Bight (23° 37′S to 23° 44′S and 45° 24′W to 45° 26′W) is about 16 km long and contains several sandy beaches. Two homogeneous areas, i.e., two areas that are some distance apart but have as similar features as possible, named South and North, each 2.0 × 0.8 km in extent, were selected so as to avoid as much as possible the influence of rivers entering the bight (for more detail see [28]). This procedure better represented the whole bight, particularly shallow sites with relatively little continental influence. The South area extends from Porto Novo to Palmeiras beaches; it has a gentler slope, and is on the side of the bight where the Juqueriquerê River, the largest river, enters the bight. The North area, between Indaiá and Centro beaches, has a steeper slope and is on the side where the smaller Lagoa and Santo Antônio rivers enter the bight.

### Sampling procedures

Sampling was performed monthly from August 2003 through October 2004, under license from the appropriate federal environmental agency (IBAMA-DIREN No. 08/2001), in Caraguatatuba Bight. In each area, the beach length of 2000 m was divided into 10-m intervals, and three sampling stations were randomly selected from these 200 possibilities in each month. The position of the station was stored in a GPS at MLW (mean low water). From this point, a fishing boat (class G2M, 11 m long with a 22-HP engine) performed 800-m trawls perpendicular to the beach, starting at a point 400 to 1600 m distant from the MLW line. This interval is equivalent to depths from 1 to 4 m. The trawling speed was 1 kt, and the trawl was performed with a bottom otter trawl with 2.0 cm mesh, mouth aperture 1.6 m high and 6.0 m long, and bag depth 3.5 m.

The fish were then removed from the net and immediately preserved in a 10% formalin solution in order to paralyze the enzyme action, preserving the digestive-tube contents. After sorting and identification, the specimens were preserved in 70% ethanol, and then measured (total and standard length, cm). A total of 100 individuals (25 for each season) were selected from the total of 5970 individuals obtained over the sampling period, with the use of a random-digits table. An abdominal-ventro-sagittal incision was made from the anal aperture to the pelvic-fin insertion, and the digestive tube was removed and its length measured (from the beginning of the esophagus to the end of the rectum; DTL). The contents of each digestive tube were identified and the volume of each group in each stomach was measured. The nematodes found in the digestive tract were considered to be parasites rather than food items, because they showed no sign of digestion.

### Data analysis

The mean number and size of individuals (+SE) of *C. spixii* were calculated for months and areas (South and North). Two-way ANOVAs (Area × Month) were performed to test the distribution patterns and were replaced by the equivalent non-parametric tests when necessary, i.e., Sheirer-Ray-Hare two-way ANOVA of ranks (for abundance, equal sample sizes in each cell) and Kruskal-Wallis and Mann-Whitney for size distribution (months and areas, respectively). The relationship between total and standard length was calculated through a linear regression. Standard length was used to perform all further analyses.

Growth parameters were estimated based on von Bertalanffy growth curves, by using the FISAT software and following the methodology proposed by the ELEFAN I and Shepherd′s routines, and were combined in a general K-scan [29], to search for a common peak of optimum goodness-of-fit, before refining the parameter analysis. Because the length of the largest individual collected was below the maximum length recorded in previous studies (30 cm total length; [6], [25]), literature values were used as the basis to define an asymptotic length range for the purpose of the analysis. We assumed a range of values, for the asymptotic length, between the maximum recorded in the literature (∼30 cm TL) and its division by 0.95 [30]. This latter value was rounded up to slightly increase the range of values; the former was not rounded up, since a value lower than a previously observed length does not seem to fit the definition “infinitely old”. Since literature values refer to species' total length (TL), we used the regression fit for our sample to adapt these base values for the calculation of the asymptotic length.

Based on the values determined as above, the total mortality index (Z) was estimated through the use of the length-converted catch curve (the rate at which the number of individuals decreases over time), by the slope of the linear regression: log_e_(N/Δt) = *a*+*b*t; where N is the number of individuals in a given length class, Δt is the time needed for the fish to grow through this length class, t is the mean age of individuals in this length class, *a* is the regression intercept, and *b*, with the sign changed, is the estimate of Z [31].

In the dietary analysis, for each stomach item the frequency of occurrence (FO) and percent volume (V) were calculated, where FO was calculated as the percentage of stomachs containing food, and V was calculated by dividing the volume of each food category by the weight of all items in the respective digestive tract. Because of the fragmentation of the items in the digestive tube, the numerical composition was not calculated. The amount of unidentified (n.i.) content (organic matter, OM) was apportioned among the identified items. The values obtained were then graphed, as proposed by Costello [30], to calculate an index of dietary importance (IAi) as proposed by Kawakami and Vazzoler [32]: IAi = FOi*Vi/Σ (FOi*Vi). This index was then used to calculate the similarity index (PS) proposed by Schoener [33] and Feinsinger et al. [34], to compare the similarity among seasons, described by the formula: PS = 1−(0.5*Σ|IAi_x_−IAi_yi_|), where *IAixi* is the index of dietary importance of item *i* in season *x* and *IAiyi* is the index of dietary importance of item *i* in season *y*.

## Results

**Figure 1:**
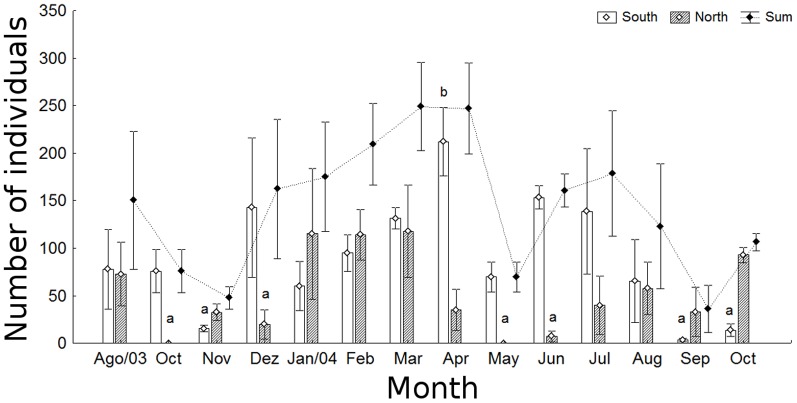
Spatial and temporal variation in number of individuals. Spatial and temporal variation in the number of individuals (mean±standard error; α = 0.05) of *Cathorops spixii* in Caraguatatuba Bight. The fish were sampled from August 2003 through October 2004. Different letters denote significant differences among months and areas, discriminated by the SNK test; absence of letters indicates no difference from any other value.

The density of *C. spixii* individuals was noticeably higher in the South area. Collections from the North area outnumbered those from the South in only 5 of the 14 months (Figure 1). In general, the abundance of *C. spixii* decreased from August to November 2003 and then increased from December on, reaching the highest levels in March and April 2004. An abrupt decrease was observed in May, and then a second peak of abundance in July. For the temporal and spatial changes in abundance, the non-parametric Scheirer-Ray-Hare test indicated a significant interaction between the factors time and space (H = 24.51, d.f. = 13; p<0.05); no significant differences were found among months (H = 19.89, d.f. = 13; p = 0.098) but were found between areas (H = 6.96, d.f. = 1; p<0.01), i.e., density was higher in the South area. The interaction, however, indicated that the largest numbers of fish were concentrated in the South area in April 2004; and the smallest numbers were observed in both the South (in November 2003, September and October 2004) and North (October 2003, December 2003, May and June 2004) areas (Figure 1).

**Figure 2:**
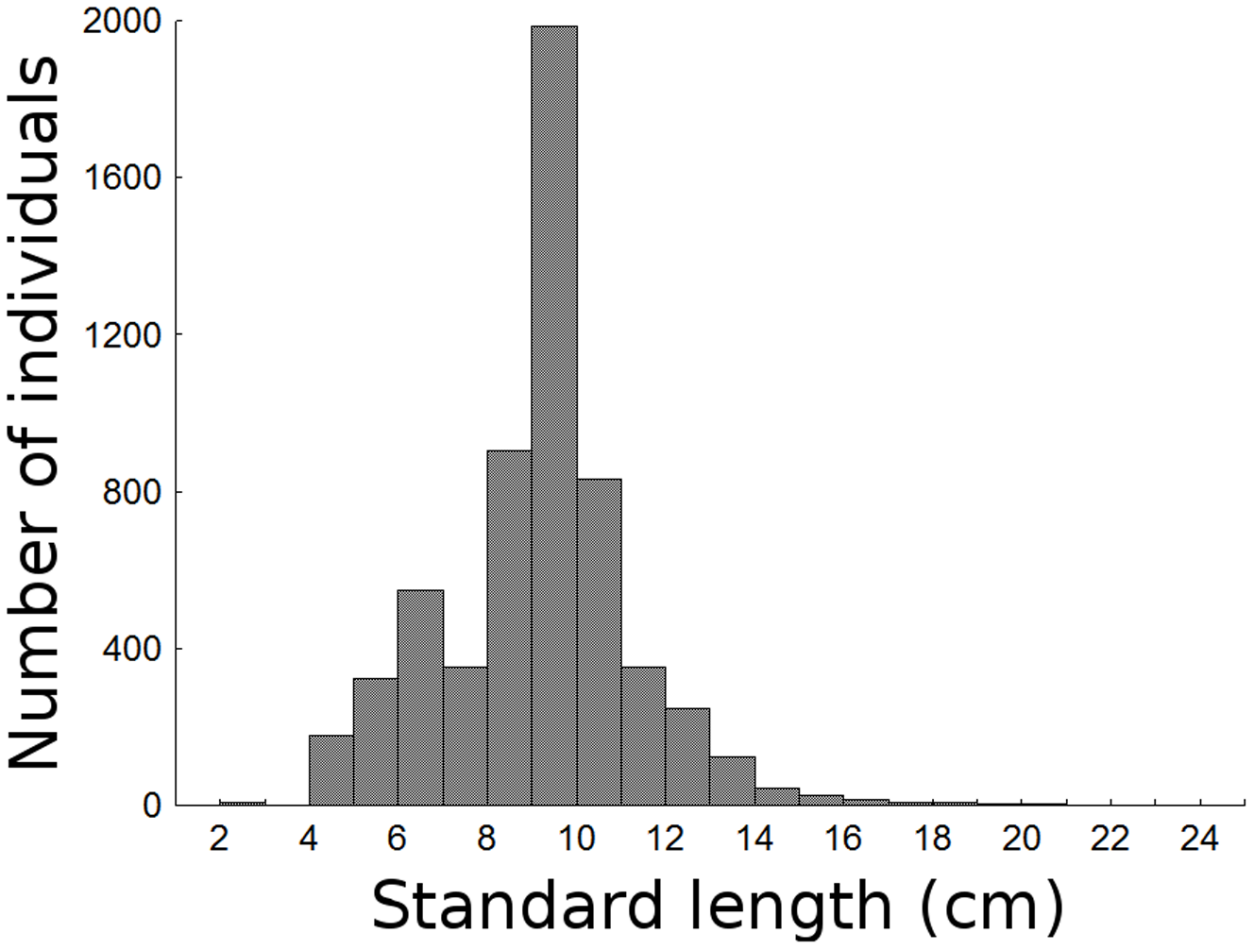
Length-class distribution. Total length- (cm) class distribution for *Cathorops spixii* sampled in Caraguatatuba Bight from August 2003 through October 2004.

**Figure 3:**
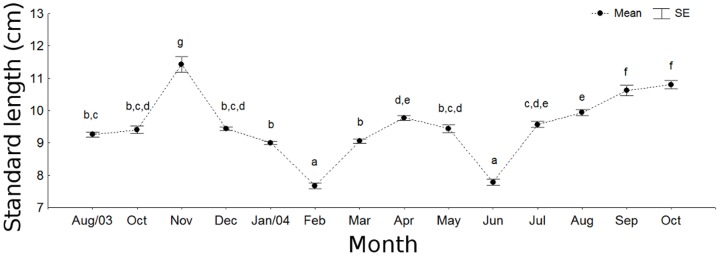
Temporal length variation. Temporal variation of standard length (cm) (mean±standard error; α = 0.05) for *Cathorops spixii* sampled in Caraguatatuba Bight from August 2003 through October 2004. Different letters denote significant differences among months, discriminated by the SNK test; brackets on the x-axis include the number of observations in each case.

**Figure 4:**
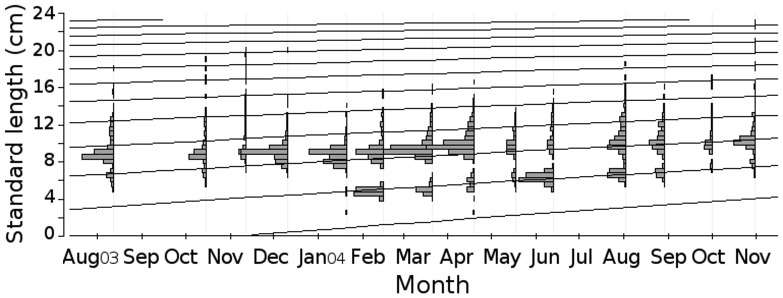
Body-growth function adjusted for *C. spixii*. Von Bertalanffy body-growth function for *Cathorops spixii* individuals sampled in Caraguatatuba Bight from August 2003 through October 2004, using the ELEFAN I routine. L∞ (maximum length)  =  27.3; K (growth-curve parameter)  =  0.16; Rn (goodness of fit) = 0.259.

The relationship between the observed total (TL) and standard length (SL) was described by the formula: TL = 1.15*SL+0.51 (cm; 95% CI; p<0.01; r^2^ = 0.991). The number of individuals measuring 13 cm (SL) and longer was notably small during the entire sampling period. A bimodal composition was observed, with a small mode of individuals around 6.5 cm and a larger mode around 9.5 cm (Figure 2). There were significant differences in the size distribution between areas (U = 3398091; d.f. = 5968; p<0.01), with fish in the North area showing larger sizes (9.70 cm±1.95 SE) than the South (8.98 cm±2.24 SE). There were also significant differences in the size distribution over the sampling period (H = 1067.57; d.f. = 13; p<0.01; Figure 3). The biological meaning of these statistical differences in size distribution must be carefully considered (because not all months and areas had sampled individuals pseudoreplicates had to be used). Notwithstanding, size fluctuation over time is consistent with the sequence of the main modes (Figure 4): a general main, higher mode, which remained fairly constant over time, shifted slightly to the right from August to November 2003, so that this last month showed one of the highest mean values of the entire study period. In December the tail of larger individuals that was observed previously, virtually disappeared, leading to a decrease in mean length. This situation persisted in January, when a discrete entry of smaller individuals was noted. From February 2004 on, the number of smaller individuals increased and formed a new mode (around 4 cm standard length), which gradually shifted to the right in the succeeding months. Consequently, February 2004 showed an abrupt decrease in mean length. The same was observed in June 2004, when the lower mode again exceeded the higher one. In the other months, the gradual shift to the right of the lower mode led to intermediate, and increasing, mean values over time.

The increase in size of the lower modes indicated a slow growth of *C. spixii* individuals. In agreement, VBGF generated low values of K. Asymptotic length was 27.3 cm (SL) and K was determined as 0.16 yr^−1^, corresponding to an Rn of 0.259. The lower mode contributed greatly to the reliability of the growth function, because the constancy of the higher mode over time would be more likely to bias this analysis. The calculated t_0_ value was −1.09 yr and t_max_ was 17.63 yr. Total mortality (Z) was estimated as 1.01 yr^−1^.

**Figure 5:**
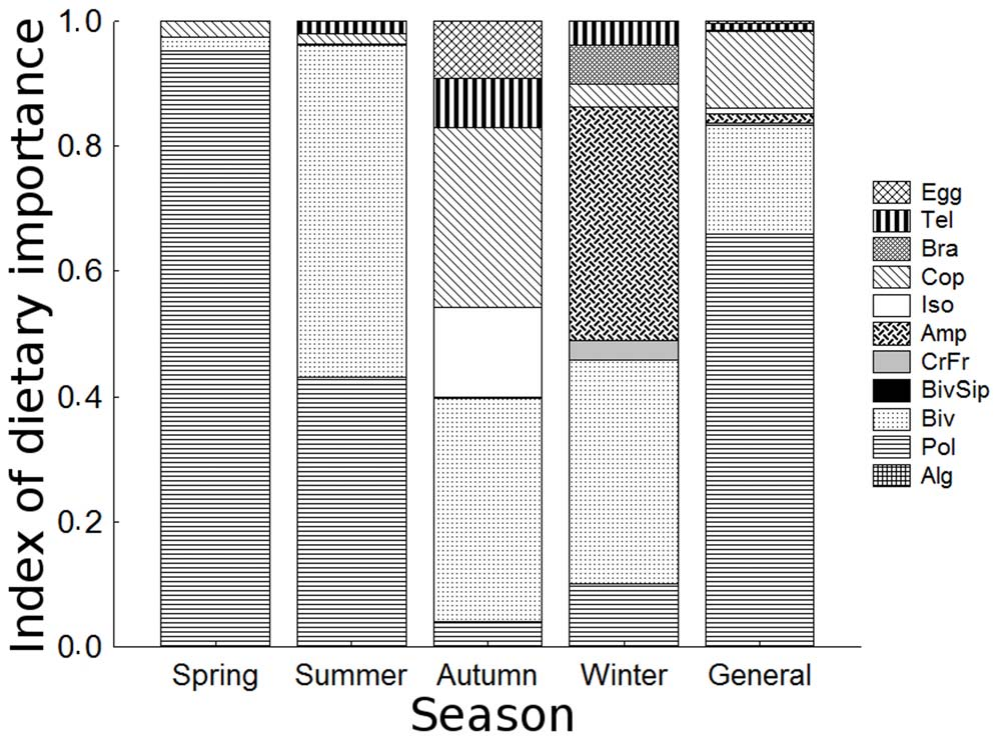
Index of dietary importance for each prey group. Index of dietary importance (IAi) for each prey group found in the stomach contents of *Cathorops spixii* collected in Caraguatatuba Bight from August 2003 through October 2004. Values are given by season and overall. **Abbrev.**: Polychaeta (Pol), Bivalvia (Biv), Copepoda (Cop), Amphipoda (Amp), Isopoda (Iso), Teleostei (Tel), unidentified crustacean fragments. (CrFr), Bivalvia siphons (BivSip), Algae (Alg).

The relationship between the digestive tube length and the standard length (DTL:SL) of *C. spixii* resulted in the following regression equation: DTL = –1.84*SL–5.48 (cm; 95% CI; p<0.01; r^2^ = 0.962), for a SL ranging from 5.6 to 15.5 cm. These catfish had a diverse diet composed of 12 different prey groups, ranging from algae to fish. The most important groups were Polychaeta, Bivalvia and Copepoda. Bivalve siphons were treated separately from other remains of this group, because siphons may result from grazing rather than from predation. The overall importance values (IAi) indicated a major importance of Polychaeta and a lower and more equal importance of Bivalvia and Copepoda (Figure 5). However, the relative importances varied over the study period.

**Figure 6:**
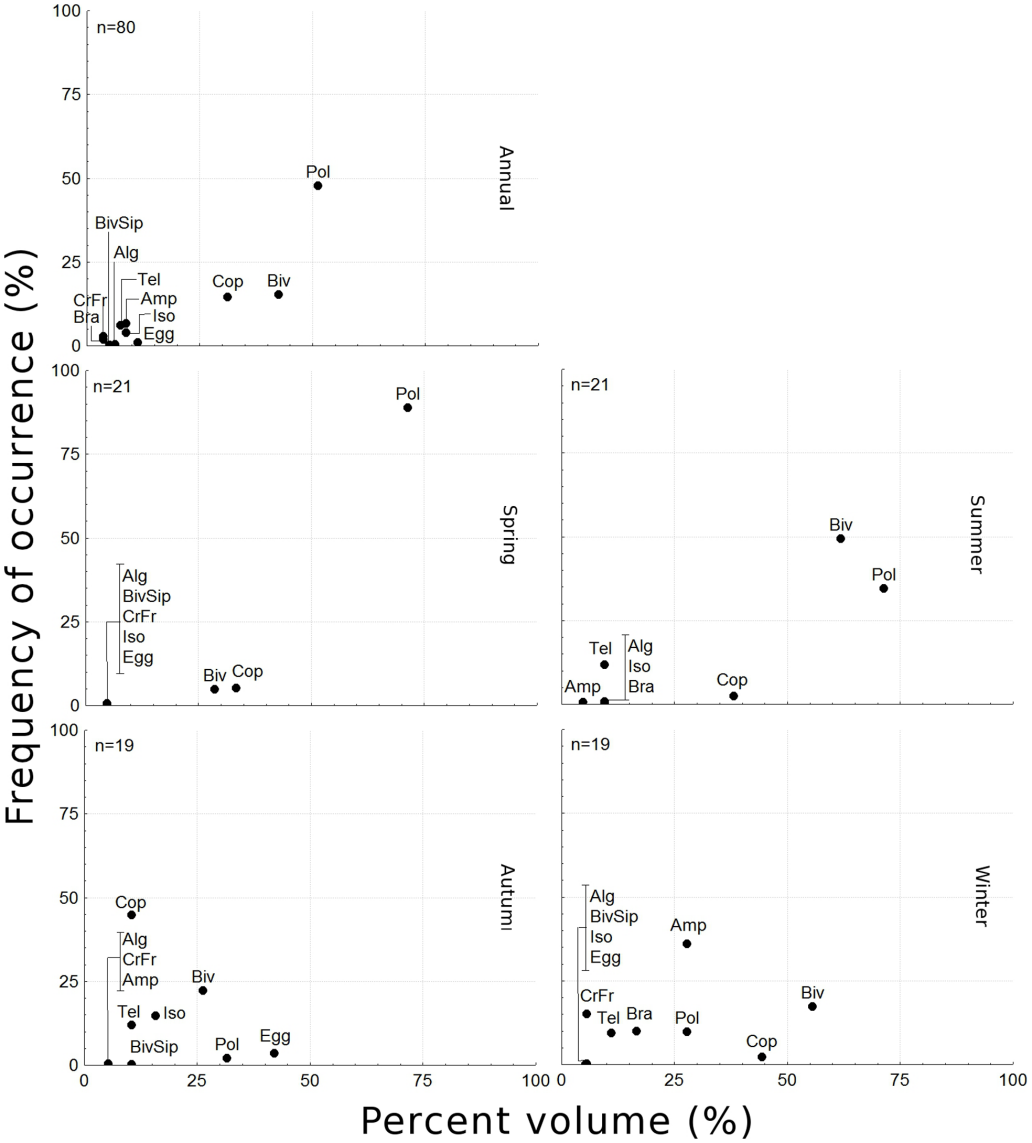
Weight versus frequency of occurrence of each prey group. Weight percentage (W%) and frequency of occurrence (FO) of each prey group found in the stomach contents of *Cathorops spixii* collected in Caraguatatuba Bight from August 2003 through October 2004. Composition is shown by season and overall. **Abbrev.**: Polychaeta (Pol), Bivalvia (Biv), Copepoda (Cop), Amphipoda (Amp), Isopoda (Iso), Teleostei (Tel), unidentified crustacean fragments. (CrFr), Bivalvia siphons (BivSip), Algae (Alg).

Polychaeta reached their highest importance in spring, with Bivalvia and Copepoda next in importance because of their FO; V% was very low for both these groups as well as for other ingested items (Figure 6). In summer, Polychaeta decreased in V% but its FO remained steady. Bivalvia, in contrast, increased significantly in both parameters. The values for Copepoda remained approximately the same, and so did those of other items. In autumn the fish showed a more generalist diet and therefore more equally distributed values of importance among items. In this season, Polychaeta had its lowest importance, and Bivalvia also decreased in both FO and V%. A few individuals of *C. spixii* ingested large amounts of copepods, raising the importance of this group, so that its FO was even lower than in the other seasons. Other items including eggs, teleost scales and other small crustaceans showed increases in one or both parameters. Winter was highlighted by the increase in importance of Amphipoda, and the re-increase in FO of Bivalvia, although V% decreased slightly. Polychaeta and Copepoda continued with low values, and were distinguished from the less-important items only due to their FO. Therefore, similarity values were low, ranging from 0.09 to 0.47 (Table 1). The lowest values were those comparing spring with both autumn and winter.

**Table:** Table 1. Matrix of similarity indexes (PS) between seasons obtained for the main prey groups that constituted the diet of Cathorops spixii collected in Caraguatatuba Bight from August 2003 through October 2004.

Seasons	Spring	Summer	Autumn
Summer	46.73		
Autumn	8.63	43.48	
Winter	14.71	49.46	47.37

Values range from 0 to 100.

## Discussion

*Cathorops spixii* has been reported from a wide variety of environments, and is generally thought to use estuaries as nursery areas for both spawning and growth [22], [25]; although some studies indicate that these areas are not necessarily used for reproduction [35]. The data from the present study revealed some variation in the abundance of the *C. spixii* population over the study period and areas. A peak in the number of individuals in March and April 2004 was also observed for other species in the area (data not shown), indicating that this feature of the population may have been linked to a major event in the area, which would have attracted a wider range of the surrounding ichthyofauna to this part of the bight.

The predominance of individuals shorter than 10 cm (SL) in the area is interesting. The area is not estuarine, but apparently still functions as a nursery for individuals in a late stage of development. The constancy of individuals of a similar size range (9.5 cm mode) throughout the sampling period is most likely to result from the presence of different cohorts. This species' smallest recorded size at first maturity is 9.5 cm SL [18], [20]. Rueda and Defeo [7], in Colombia, found virtually no mature individual of *C. spixii* under 15 cm in length (TL), which corresponds to approximately 12.5 cm SL according to the estimates for the present population.

A possible explanation is that larger individuals are more often caught by shrimpers as bycatch. Because the biomass of the ichthyofauna is more than four times that of the shrimp caught [36], this pressure is very likely to occur to some extent, but is not enough to explain the population structure observed here. A sufficiently strong pressure on larger (mature) individuals to nearly eliminate them is consistent with the large contingent of younger individuals present in the area. Furthermore, the absence of individuals smaller than 4 cm during most of the sampling period cannot be explained by invoking the same net selectivity. Therefore, if the individuals found in this area were not underdeveloped – the species can reach 30 cm in total length - the reproductively active individuals of this population must be located elsewhere.

An important feature for the smaller individuals is the entry of younger ones, about 4 cm SL, into the area, by February 2004. This cohort provided more-reliable information about growth parameters, because it was clearly distinguishable from the others, which remained more constant in the area. The results indicate that the growth of *C. spixii* is slow (K = 0.16), as also described by Taylor and Menezes [36]. Interestingly, this event agrees with the reproduction period described by other authors for tropical areas, i.e., mainly during spring and early summer [16], [17], in that they started entering the study area in late summer, at about 4 cm SL. Tíjaro et al. [37] found a growth constant of 0.38 yr^−1^, but with fairly low values of Rn, because their samples lacked virtually any modal progression and were basically constituted of older individuals (10 cm and longer). Probably many other studies have dealt with similar situations, mainly in tropical environments. For example, Garcia and Duarte [38], although they sampled large numbers of *C. spixii* individuals, found that their data were not sufficiently reliable to allow the estimation of body-growth parameters.

Therefore, although estimates of parameters may closely approximate the real values, it is necessary to carefully consider population features during the analysis and also after its conclusion, especially for management purposes. Here, the entry of a younger cohort, represented by the smaller mode, provided important information for the estimation of body-growth parameters, with a reasonable value of Rn. However, the high growth rate of this young cohort, along with the low occurrence of larger individuals in the study area must be taken into account. The high longevity estimated through this model, along with a low growth constant, concords with the literature information and with other K-strategist characteristics of this species [39]. In turn, if older individuals are leaving the area, this implies that the mortality rate includes the number of individuals that actually out-migrated, and which would, in this case, be the major factor responsible for the high value obtained.

The DTL/SL was higher than 1.0 for all individuals, whatever their size, which concords with the omnivorous feeding habit also observed through the prey composition. These fish took prey from several trophic levels, but mainly, except for Polychaeta, from lower ones, i.e., Bivalvia, Copepoda, detritus, and algae, thus placing them at a low trophic level relative to other fishes in the bight. Concerning the main items, the predominance of Polychaeta in the diet of *C. spixii* was reported previously [27], but other studies have also found crustaceans, mainly copepods and amphipods, as the main food item in the diet of this catfish [23], [24]. Here, although still a large part of the diet, copepods were the third most important item, and amphipods were the fourth. The second most important item found here, Bivalvia, did not reach considerable importance values in other studies [19], [23], [27]. Espírito-Santo and Isaac [24] cited Bivalvia as an important item for individuals of *C. spixii* longer than 10 cm in the Caeté River (Pará, Brazil). A reasonable explanation for the particularly high proportion of Bivalvia observed here may be the locally high abundance of *Tivela mactroides* [40], along with the opportunistic characteristics of this catfish [27].

The small amounts of bivalve siphons and fish scales ingested suggest that this species also behaves as a grazer, but it is more likely that they represent incidental ingestions, which matches its bottom-foraging behavior. Also, the ingestion of scales may be due to sampling artifacts. Even though they intentionally prey on scales - there are reports of ingestion of scales by ariids, mainly juveniles [41] – it is not known if this results from lepidophagy or necrophagy.

A large amount of unidentified organic matter was found in the digestive tract of *C. spixii*. This material was not included in the analysis because it provides no useful information on prey composition. However, as organic matter comprised over 90% of the total amount of food, it is unlikely to be due only to accidental ingestion from bottom foraging. Rather, this may indicate some other feeding habits of the fish, such as a preferred foraging period. Because all the samples were taken in daytime, this feature could be related to a nocturnal foraging habit, although no studies have yet examined this issue. Other studies did not find significant amounts or did not mention the presence of organic matter in the diet of *C. spixii* [19], [23], [24]. The scatterplots indicate that they are largely generalists, although changes in food availability may allow them to consume larger amounts of one or another item at different times, and the pronounced seasonality observed is likely also a factor.

Environmental conditions affecting the local bottom communities would, therefore, be the factor most responsible for the observed low percentage of similarity between seasons. Melo and Teixeira [19] found large numbers of polychaetes in the diet of the marine catfishes *Arius rugispinis* and *C. spixii* during the dry season in Maceió, Alagoas, northeastern Brazil, contrasting with higher proportions of crustaceans and fish scales during the rainy season. These differences were attributed to the hypoxic conditions in the area during the dry season, and therefore the items ingested were also attributed to their availability rather than to a preference of the predator itself.

Chalom et al. [27], studying estuarine areas, found, in addition to seasonality, ontogenetic variation in the diet of this catfish. Similarly, Díaz and Bashirullah [23] found that younger individuals of *C. spixii* have a more diverse diet than larger ones. Changes in dietary habits during development are commonplace and are related to an increasing efficiency of energy intake, due to their size relative to their prey and to changes in the morphology of the digestive tract and oral apparatus. This pattern agrees with the data for population biology observed here, because the study area, although it has more oceanic than estuarine characteristics, harbored primarily young individuals.

Taken together, this information indicates that the bight as a whole is important for the development of this catfish; one prominent peak of recruitment was observed throughout the year (and was important to guide the analysis of body growth parameters); the body-growth constant was consistent with a K-strategist species; individuals in this area showed an opportunistic feeding habit, preying mainly on low trophic levels. However, given the lack of older individuals, which probably migrated to other areas, these features must be carefully considered for extrapolations and comparisons to other areas/populations. The specific features of the population in this area must be taken into account for management purposes.
